# Cyclodextrin-mediated improvement of red wine color quality dissected by coloromics and anthocyanin evolution dynamics

**DOI:** 10.1016/j.fochx.2026.104199

**Published:** 2026-07-14

**Authors:** Caiyun Liu, Zengshuai Zhang, Jing Li, Xiaoyu Zhang, Mario Prejanò, Tiziana Marino, Yongsheng Tao, Yunkui Li

**Affiliations:** a College of Enology, Northwest A&F University, Yangling 712100, China; b Dipartimento di Chimica e Tecnologie Chimiche, Università della Calabria, Arcavacata di Rende, CS, Italy

**Keywords:** Cyclodextrins, Anthocyanin derivatives, Pyranoanthocyanins, Wine color evolution, Targeted metabolomics, Chemometric analysis

## Abstract

This study elucidated the mechanisms by which pre-fermentation addition of cyclodextrins (CDs) regulates anthocyanin transformations and wine color evolution using a mechanistic coloromics approach. Musts were treated with α-CD, β-CD, and HP-β-CD, and 79 anthocyanins across 11 structural categories were profiled to characterize pigment pathways. Multivariate chemometric analyses integrating pigment composition with color and UV–Vis spectral parameters revealed distinct regulatory effects. α-CD accelerated the depletion of monomeric anthocyanins and promoted their conversion into vitisin-type pyranoanthocyanins, enhancing red hue intensity and chromatic evolution. In contrast, β-CD and HP-β-CD slowed monomer conversion and facilitated gradual formation of derived pigments, improving long-term color stability. Chemometric analyses (PCA, PLSR, Mantel test, and CCA) further indicated that CD cavity size and flexibility selectively stabilize anthocyanins and intermediates, thereby modulating pigment formation. Overall, α-CD acts as an active kinetic regulator that channels unstable free anthocyanins into resilient vitisin-type pyranoanthocyanin networks, while β-CD and HP-β-CD function as passive stabilizers through steric shielding of monomeric and acylated anthocyanins, together providing a strategy to improve wine color quality.

## Highlights

• Mechanistic coloromics reveals how pigment dynamics drive color evolution.
• CDs selectively stabilizes phenolics, indirectly modulating anthocyanin dynamics.
• α-CD promotes early formation of VitB and EPyA–F, intensifying red hue.
• β-/HP-β-CD protect monomers and acylated anthocyanins, regulating derivatives.
• Addition of CDs provide a mechanism-based strategy to enhance wine color quality.

## Graphical abstract

**Figure:**
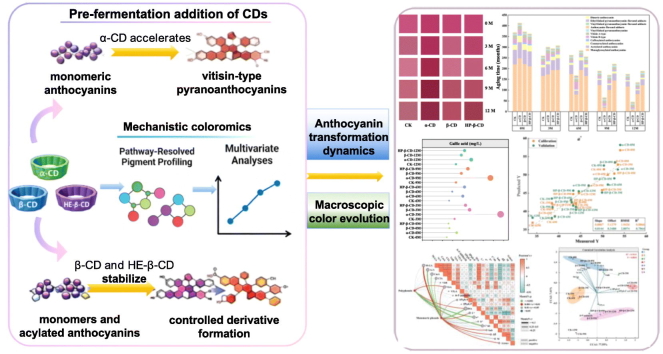

## 1 Introduction

Color is one of the most critical quality attributes of red wine, directly influencing consumer perception, market value, and sensory acceptance. Among the key determinants of wine color, anthocyanins and their derivatives play a central role in defining red hue, brightness, and long-term color stability (Wu, Zhang, et al., 2025; Zhang et al., 2020; Wu, Zhang, et al., 2025). However, despite extensive characterization of anthocyanin composition and copigmentation mechanisms, achieving and maintaining desirable color quality and stability during wine aging remains a persistent challenge in enology (Cheng et al., 2023; Wu, Prejanò, et al., 2025). This challenge is primarily manifested by age-related anthocyanin degradation, premature color loss, and unpredictable color evolution, particularly in wines intended for extended aging.

Anthocyanins in wine undergo complex transformation pathways during fermentation and aging, including hydration, polymerization, condensation with flavanols, and conversion into more stable pyranoanthocyanin derivatives such as vitisin-type pigments (Huang et al., 2024). Collectively, these transformations shape wine color evolution, determining whether color development is rapid but unstable or gradual and persistent. While numerous studies have focused on individual anthocyanin derivatives or isolated reactions, how competing transformation pathways are dynamically balanced and linked to macroscopic color evolution remains poorly understood (Dangles, 2024). This lack of mechanistic insight limits the ability to predict or mechanistically control wine color evolution. From an industry perspective, current strategies to improve wine color stability remain largely empirical. Practices such as maceration management, oxygen control, tannin addition, and copigment supplementation are widely employed (Wu, Zhang, Prejanò, et al., 2024). However, their effects are highly variable across grape varieties, vintages, and winemaking conditions (Liu, Zheng, et al., 2025; Longo et al., 2021; Wu, Zhang, Fan, et al., 2024). More importantly, these approaches primarily target downstream color dynamics rather than the upstream molecular kinetics governing anthocyanin evolution (Wu, Zhang, Fan, et al., 2024). As a result, winemakers still lack reliable, mechanism-based tools to improve color quality in a predictable and controllable manner.

Cyclodextrins (CDs) are cyclic oligosaccharides characterized by hydrophobic cavities and hydrophilic outer surfaces, enabling the formation of inclusion complexes with a wide range of compounds. Although CDs are widely applied in food and pharmaceutical systems, their application in winemaking remains limited (Astray et al., 2009). Our previous studies have demonstrated that CDs can protect aroma compounds, improve sensory quality, and stabilize phenolic constituents in wine (Liu, Wei, et al., 2025). Moreover, their unique cavity sizes and molecular flexibility enable selective interactions with small phenolics, pigments, and reactive intermediates, highlighting a promising yet underexplored potential to modulate anthocyanin dynamics in red wine (Liu, Wu, et al., 2024). A key unresolved question is whether CDs can be applied not merely as passive stabilizers, but as active kinetic regulators that channel anthocyanin transformation toward distinct chromatic outcomes, thereby modulating pigment transformation pathways toward desired color dynamics. Specifically, it remains unclear how different CD types, α-CD, β-CD, and hydroxypropyl-β-cyclodextrin (HP-β-CD), interact with monomeric anthocyanins, acylated anthocyanins, and phenolic cofactors, and how these interactions translate into distinct chromatic trajectories during wine aging.

To overcome the limitations of current wine color research, an integrative and mechanistic framework is required to elucidate the dynamics relationships between anthocyanin transformation and macroscopic color evolution during wine aging. Existing research largely examines bulk color parameters or a narrow subset of anthocyanins (Bueno et al., 2012). By ignoring the interconnected and competing nature of pigment transformation pathways, these methods provide limited understanding of the mechanisms underlying dynamic color evolution (Trouillas et al., 2016). Mechanistic coloromics integrates pathway-resolved pigment profiling with multivariate analyses to link anthocyanin transformation dynamics to macroscopic color evolution, providing a framework to evaluate how CDs modulate color trajectories (Long et al., 2026). This approach enables quantitative tracking of pathway-specific pigment dynamics and their temporal contributions to macroscopic color evolution, allowing the identification of dominant reaction routes, critical intermediates, and rate-limiting steps. Importantly, it distinguishes between pigment stabilization and pigment transformation. This provides a framework to evaluate whether CD addition affects wine color primarily by preserving existing anthocyanins or by redirecting pigment transformations along competing pathways.

In this study, mechanistic coloromics was employed to elucidate how the pre-fermentation addition of structurally distinct CDs regulates anthocyanin evolution dynamics, thereby improving wine color quality and stability during aging. Musts were separately supplemented with α-CD, β-CD, or HP-β-CD prior to fermentation, and 79 anthocyanins and their derivatives across 11 structural categories were quantitatively monitored throughout aging, enabling pathway-level dissection of pigment dynamics. Multivariate analyses—including principal component analysis (PCA), partial least squares regression (PLSR), Mantel correlation, and canonical correspondence analysis (CCA)—integrated CD structural features, anthocyanin dynamics, and color parameters, capturing the coupled molecular and macroscopic processes underlying wine color evolution. By comparing CDs with distinct cavity sizes and molecular flexibilities, this study demonstrates how selective stabilization of monomeric, acylated, and intermediate anthocyanins regulates pigment kinetics and directs macroscopic color evolution. Overall, this work elucidates the mechanisms by which CD structure governs anthocyanin dynamics and wine color evolution, providing a novel, mechanism-driven strategy for the practical modulation of red wine color during aging.

## 2 Materials and methods

### 2.1 Materials

Grapes: Cabernet Sauvignon grapes were harvested in October 2023 from the wine experimental and demonstration station of Northwest A&F University, located in the eastern foothills of the Helan Mountains, Minning, Ningxia Province, China (E106°05′, N38°56′).

Yeast: The commercial yeast *Saccharomyces cerevisiae* BV818 was supplied by Angel Yeast Co., Ltd. (Hubei, China).

Main reagents: Gallic acid (CAS 149–91-7, MW 170.12, purity ≥99.0%), caffeic acid (CAS 331–39-5, MW 180.16, purity ≥98.0%), (+)-catechin (CAS 154–23-4, MW 290.27, purity ≥97.0%), α-CD (CAS 10016–20-3, MW 972.84, purity ≥98.0%), β-CD (CAS 7585-39-9, MW 1134.98, purity ≥98.0%), and HP-β-CD (CAS 128446–35-5, MW 1541.54, purity ≥98.0%) were purchased from Shanghai Aladdin Biochemical Technology Co., Ltd. (Shanghai, China). Malvidin-3-*O*-glucoside was obtained from Shanghai ZZBIO Co., Ltd. (Shanghai, China). Pectinase was purchased from Shanghai Yuanye Biotechnology Co., Ltd. (Shanghai, China). Other reagents, including hydrochloric acid, methanol, sulfurous acid, sodium chloride, and acetaldehyde, were obtained from Solarbio Technology Co., Ltd. (Beijing, China). All chemicals and reagents used in this study were of analytical grade.

### 2.2 Methods

#### 2.2.1 Winemaking experiment

Cabernet Sauvignon grapes were manually destemmed, crushed, mixed thoroughly, and evenly distributed into four 10 L food-grade glass fermenters. Sulfur dioxide (SO_2_, 60 mg/L) was added immediately after crushing, followed by the addition of pectinase (20 mg/L) after 2 h. Subsequently, α-CD, β-CD, or HP-β-CD was individually added to three fermenters at a final concentration of 3 g/L, while the remaining fermenter served as the control. After 48 h of cold maceration at 4 °C, *Saccharomyces cerevisiae* BV818 was inoculated at 200 mg/L to initiate alcoholic fermentation once the temperature increased to 25 °C. Fermentation progress was monitored by measuring temperature and specific density. When residual sugar decreased below 4 g/L, alcoholic fermentation was terminated by the addition of 60 mg/L SO_2_. Following fermentation, the wines were divided into five portions and transferred into 750 mL brown glass bottles to minimize light exposure during aging. One portion was analyzed immediately, while the remaining samples were aged for 3, 6, 9, and 12 months, respectively. All bottles were stored in a temperature-controlled wine cellar maintained at approximately 14 °C with appropriate humidity at the College of Enology, Northwest A&F University (Yangling, China) until analysis.

#### 2.2.2 Determination of CIELAB color parameters

Wine color was quantified using the CIELAB system. The visible spectra (400–780 nm) of each sample were recorded at 1 nm intervals with a Cary 60 UV–Vis spectrophotometer (Agilent Technologies, USA). All measurements were performed in a 2 mm quartz cuvette, with deionized water as a blank reference. Specific absorbance readings at 450, 520, 570, and 630 nm were employed to calculate the *L*^⁎^, *a*^⁎^, *b*^⁎^, C* ab, and *h*_ab_ according to a 10° standard observer and D65 illuminant (Wu, Zhang, Fan, et al., 2024). Differences in color between samples (Δ*E*) were computed as the Euclidean distance within the three-dimensional CIELAB space (Fan et al., 2023).

#### 2.2.3 Determination of UV–vis spectral parameters

The absorbance at 520 nm and the wavelength of maximum absorption (*λ*_max_) were recorded from the UV–Vis spectra of wine samples scanned between 400 and 800 nm (Liu, Wei, et al., 2025). The relative absorbance change at 520 nm (*M*) and the shift in λ_max_ (Δ*λ*_max_) were calculated according to the following equations:

(1) $$M\;\left(\%\right)=\frac{A-A_{0}}{A_{0}}\times 100\%$$

where *M* represents the relative absorbance change at 520 nm (%), and *A* and *A*_0_ represent the absorbance values at 520 nm of the CD-treated wine and the corresponding control wine without CD addition, respectively.

(2) $$\Delta \lambda_{\max}=\lambda_{\max}–\lambda_{\text{max0}}$$

where Δλmax represents the shift in the wavelength of maximum absorption, and *λ*_max_ and *λ*_max0_ represent the maximum absorption wavelengths of the CD-treated wine and the corresponding control wine without CD addition, respectively.

#### 2.2.4 Determination of non-anthocyanin phenolic components

Monomeric phenols were extracted by combining 2 mL of wine with 2 mL of ethyl acetate and 1 mL of acetonitrile, followed by brief vortexing for 10 s. The mixture was then centrifuged at 8000 rpm for 15 min (HC-30182; Anhui Zhongke Zhongjia Scientific Co., Ltd., China), and the upper layer was collected. The extraction procedure was repeated twice, and the pooled organic fractions were evaporated under reduced pressure using a rotary evaporator. The resulting residue was reconstituted in 1.0 mL of methanol before high-performance liquid chromatography (HPLC; Shimadzu, Kyoto, Japan).

Monomeric phenols were analyzed by HPLC using a Synergi Hydro-RP C18 column (250 × 4.6 mm, 4 μm; Phenomenex, Torrance, CA, USA). The mobile phase consisted of acetonitrile and aqueous acetic acid, with Phase A containing 800 mL water, 100 mL acetonitrile, and 1 mL acetic acid, and Phase B containing 400 mL water, 500 mL acetonitrile, and 1 mL acetic acid. Gradient elution for Phase B was performed as follows: 0–35% (0–45 min), 35–100% (45–50 min), held at 100% (50–55 min), returned to 0% (55–56 min), and equilibrated at 0% (56–62 min). The injection volume was 20 μL, the column temperature was 30 °C, and the flow rate was 1 mL/min. Quantification was carried out using the external standard method (Liu, Wu, et al., 2025).

Total phenols, total flavonoids, and total anthocyanins were determined according to Liu, Wu, et al. (2024), while total tannins were measured using the vanillin assay. Total flavanols were quantified using *p*-dimethylaminocinnamaldehyde (p-DMACA) at 640 nm, following the procedure described by Liu, Gao, et al. (2024). All measurements were performed in triplicate, and results are presented as mean ± standard deviation.

#### 2.2.5 Determination of proportions of anthocyanin forms

To determine the proportions of different anthocyanin forms, 2 mL of wine was first treated with 20 μL of 10% (*v*/v) acetaldehyde and incubated for 45 min at 25 °C, after which the absorbance at 520 nm (*A*_acet_) was recorded. In a separate assay, 160 μL of 5% (*w*/*v*) SO₂ was added to 2 mL of wine, and the mixture was allowed to react for 10 min at 25 °C before measuring the absorbance at 520 nm (ASO2). Finally, wine samples were diluted 20-fold with a model wine solution (5 g/L tartaric acid, 12% v/v ethanol, 0.2 mol/L NaCl, pH 3.6) and the absorbance at 520 nm (*A*_wine_) was measured after 10 min. The free anthocyanin (*FA*%), copigmented anthocyanin (*CA*%), and polymeric anthocyanin (*PA*%) ratios were then calculated according to (3), (4), (5).

(3) $$\mathrm{CA}\%=\frac{A_{\text{acet}}-A_{\text{wine}}\times 20}{A_{\text{acet}}}\times 100$$

(4) $$\mathrm{FA}\%=\frac{A_{\text{wine}}\times 20-{A_{\mathrm{SO}}}_{2}}{A_{\text{acet}}}\times 100$$

(5) $$\mathrm{PA}\%=\frac{{A_{\mathrm{SO}}}_{2}}{A_{\text{acet}}}\times 100$$

#### 2.2.6 Determination of anthocyanins and their derivatives

Wine samples were filtered through a 0.22 μm organic membrane prior to analysis. Chromatographic separation was performed using an HPLC system directly coupled to a SCIEX TripleTOF® 5600+ quadrupole time-of-flight mass spectrometer (AB SCIEX, Singapore). Separation was achieved on a Kinetex® C18 column (100 × 2.1 mm, 1.7 μm, 100 Å; Phenomenex, Torrance, CA, USA) maintained at 55 °C. The electrospray ionization source was operated in positive ion mode (ESI+) with a spray voltage of +4.5 kV. The injection volume was 5 μL and the flow rate was 0.4 mL/min. The binary mobile phase consisted of solvent A (water/acetonitrile/formic acid, 88.9:11.1:1, *v*/v/v) and solvent B (water/acetonitrile/formic acid, 42.1:52.6:1, v/v/v). Gradient elution was programmed as follows: solvent B increased linearly from 5% to 35% (0–20 min), rapidly from 35% to 100% (20–21 min), held at 100% (21–30 min) for column washing, and returned to 5% (30–35 min) for re-equilibration. Anthocyanins and their derivatives were detected in Scheduled MRM HR mode, in which specific precursor-to-product ion transitions were monitored at high mass accuracy for simultaneous qualitative identification and quantification. The precursor-to-product ion transitions, collision energies, and reference retention times were adopted from the validated mass spectral and chromatographic database established by Zhang et al. (2020), which covers the five 3-*O*-glucoside anthocyanins, their acetylated and coumaroylated forms, vitisin-type pyranoanthocyanins, and anthocyanin-flavanol condensation products. Compound identification was based on two criteria. First, precursor-to-product ion transitions were matched to the database. The precursor ion provides information on molecular mass and structural class, whereas the product ion supports assignment of the corresponding aglycone or derivative fragment released through characteristic neutral losses (162 Da for hexose, 42 Da for acetyl, 146 Da for coumaroyl, and 72 Da for pyruvic acid). Second, the chromatographic elution order was verified against the sequence reported in Zhang et al. (2020, 2021) for C18 columns under acidic acetonitrile gradient conditions, in which compounds elute in the order of delphinidin-, cyanidin-, petunidin-, peonidin-, and malvidin-based forms within each structural category, with glucosides preceding acylated derivatives. Malvidin-3-*O*-glucoside served as the sole external calibrant for quantification. A seven-point calibration curve was constructed over the range of 0.05–50 mg/L (R^2^ ≥ 0.999), with an LOD of 0.01 mg/L and an LOQ of 0.05 mg/L determined at signal-to-noise ratios of 3 and 10, respectively. Intraday (*n* = 6) and interday (*n* = 3 days) precision, evaluated using a pooled wine sample, yielded RSDs below 3% and 5%, respectively. Matrix-matched recovery, assessed by standard addition at three concentration levels, ranged from 85% to 105% (RSD < 5%). Sample stability under autosampler conditions (4 °C, 24 h) was confirmed with peak area variation below 5%. This compound is the most abundant anthocyanin in Cabernet Sauvignon dry red wine and the primary structural precursor of the pyranoanthocyanins and anthocyanin-flavanol condensation products profiled in this study (Wang et al., 2024; Zhang et al., 2020). As authentic reference standards are not commercially available for the majority of the 81 profiled compounds, all compounds other than malvidin-3-*O*-glucoside are semi-quantified relative to this standard, and the reported concentrations represent relative abundance estimates rather than absolute values (Wang et al., 2024; Zhang et al., 2020; Zhang et al., 2021).

#### 2.2.7 Statistical analysis

All measurements were conducted in analytical triplicate, and the results are presented as mean ± standard deviation. One-way analysis of variance (ANOVA) followed by Duncan's multiple range test (*p* < 0.05) was used to evaluate differences among treatment means, using SPSS 26.0 (SPSS Inc., Chicago, IL, USA). All statistical analyses were performed using SPSS 26.0 (SPSS Inc., Chicago, IL, USA). Data visualization, correlation analysis, and principal component analysis (PCA) were performed with Origin 2021 (OriginLab, Northampton, MA, USA), with variables standardized prior to analysis. Partial least squares regression (PLSR) was carried out in The Unscrambler X 10.4 (CAMO Software, Oslo, Norway). X-variables, including anthocyanin subclasses and derivatives, were scaled by the inverse of their standard deviation (1/SDev), while Y-variables, including CIELAB color and UV–Vis spectral parameters, were assigned equal weights. The optimal number of latent variables was determined by leave-one-out cross-validation (LOO-CV; 20 segments, one sample per segment) to minimize the predicted residual sum of squares (PRESS) and reduce the risk of overfitting. Model performance was assessed using the coefficients of determination for calibration (R^2^_cal_) and cross-validation (R^2^_val_), the cross-validated explained variance (Q^2^), and the root mean square errors of calibration (RMSEC) and cross-validation (RMSECV). Mantel tests were performed using the ChiPlot online platform (https://www.chiplot.online), with Pearson's correlation as the metric, Bray-Curtis dissimilarity for the anthocyanin matrix, and Euclidean distance for the color-parameter matrix. Statistical significance was assessed by permutation testing with 1000 permutations (*p* < 0.05). Canonical correspondence analysis (CCA) was performed using the same platform, with anthocyanin data standardized by row-wise percentage scaling prior to ordination.

## 3 Results and discussion

Tracing how pre-fermentation CD addition alters wine color requires connecting the macroscopic color response to the underlying changes in phenolic composition and anthocyanin dynamics. Wine color is determined by the abundance, structural state, and transformation kinetics of anthocyanin pigments, which depend on the availability of phenolic co-reactants and on the reaction environment during fermentation and aging. The Results and Discussion therefore proceeds from characterization of color and UV–Vis spectral responses, through matrix-level examination of monomeric phenols and bulk phenolic indices, to the redistribution of anthocyanin forms and the evolution of individual pigment subclasses. Multivariate chemometric analyses are then applied to evaluate the interrelationships among these variables and to identify the pigment subclasses most closely associated with treatment-dependent color outcomes.

### 3.1 Dynamic regulation of color evolution and UV–vis spectral changes by pre-fermentation cyclodextrin addition during wine aging

**Fig. 1:**
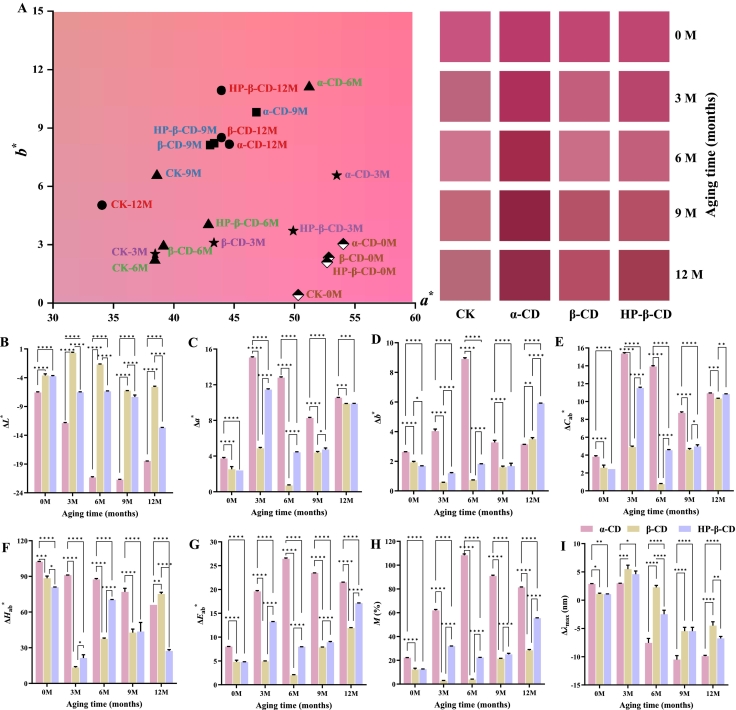
Effects of pre-fermentation addition of α-CD, β-CD, and HP-β-CD on (A) chromaticity distribution on the CIELab *a*^⁎^–*b*^⁎^ diagram (left) and corresponding color swatches (right); (B) lightness difference (Δ*L*^⁎^);(C) red-green difference (Δ*a*^⁎^);(D) yellow-blue difference (Δ*b*^⁎^);(E) chroma difference (ΔC* ab);(F) hue difference (Δ*h*_*ab*_);(G) color differences (Δ*E*), (H) hyperchromic effect (*M*), and (I) bathochromic shift (Δ*λ*_max_) during wine aging. Different letters represent significant differences (*p* < 0.05) among treatments within the same aging stage. Note: CK-0 M, α-CD-0 M, β-CD-0 M, and HP-β-CD-0 M denote the control, α-CD–treated, β-CD–treated, and HP-β-CD–treated wines at month 0 of aging, respectively. Similarly, CK-3 M, α-CD-3 M, β-CD-3 M, and HP-β-CD-3 M represent samples collected at month 3; CK-6 M, α-CD-6 M, β-CD-6 M, and HP-β-CD-6 M at month 6; CK-9 M, α-CD-9 M, β-CD-9 M, and HP-β-CD-9 M at month 9; and CK-12 M, α-CD-12 M, β-CD-12 M, and HP-β-CD-12 M at month 12. (For interpretation of the references to color in this figure legend, the reader is referred to the web version of this article.)

The influence of pre-fermentation CD addition on wine color stability was evaluated by monitoring color parameters (*L*^⁎^, *a*^⁎^, *b*^⁎^, C* ab, *h*_ab_ and Δ*E*) and UV–Vis spectral parameters (*M* and Δ*λ*_max_) over a 12-month storage period (Fig. 1). Comparison with the control revealed clear treatment-dependent differences in both the magnitude and temporal patterns of color and spectral changes, suggesting that CD type may modulate wine pigment evolution throughout aging. In terms of *L*^⁎^ (Fig. 1B), the control wine exhibited a moderate increase of 14.48% relative to the initial value at 6 months, followed by a slight decline, likely reflecting transient structural or colloidal rearrangements during mid-term aging. Wines treated with α-CD displayed a continuous decrease in *L*^⁎^, resulting in values approximately 34.72% lower than the control at 12 months. β-CD treatment resulted in a slight decrease in *L*^⁎^ of 2.89% relative to the control at 6 months, followed by a further decrease of 10.54% at 12 months, whereas HP-β-CD showed a larger decrease of 11.41% at 6 months, which progressed to a more pronounced decline of 23.81% by the end of storage. The pronounced and sustained darkening of α-CD wines relative to the control, peaking at 6–9 months, contrasts with the minimal and temporally stable *L*^⁎^ of β-CD wines throughout aging, indicating that CD structural properties determine the extent to which pre-fermentation addition influences wine color depth (Ghosh & Pal, 2007).

*a*^⁎^ exhibited pronounced differences among the wines, with CD-treated samples maintaining higher *a*^⁎^ than the control throughout the aging period (Fig. 1C). The control wine showed a rapid decrease in *a*^⁎^ of 23.57% within the first 3 months, followed by a stabilization during subsequent aging. α-CD-treated wines maintained relatively high *a*^⁎^ throughout aging, showing only a 1.00% decrease at 3 months and a 17.48% decrease at 12 months relative to their initial values, indicating a slower attenuation of red hue. β-CD and HP-β-CD treatments exhibited intermediate behaviors, with *a*^⁎^ that were 1.84% and 11.52% higher than the control at 6 months, respectively, and that increased to approximately 29% above the control by 12 months, indicating a sustained preservation of red chromaticity during long-term aging. The timing of *a*^⁎^ elevation differed substantially among treatments. α-CD wines showed the largest deviation from the control at 3 months, consistent with early-stage interactions with monomeric anthocyanins facilitated by its smaller cavity (Liu, Wu, et al., 2025). β-CD wines were nearly indistinguishable from the control at 6 months but converged toward comparable *a*^⁎^ levels as α-CD wines by 12 months, suggesting that β-CD and HP-β-CD exert their color-preserving effects primarily during long-term aging, possibly through preferential interaction with anthocyanin-derived pigments that accumulate at later stages (Wei et al., 2025).

*b*^⁎^ increased over time in both the control and CD-treated wines, indicating progressive yellowing, although the magnitude of change differed substantially among treatments (Fig. 1D). Compared with the control, α-CD-treated wines exhibited the largest increase in *b*^⁎^, reaching 5.06 times the value of the control at 6 months. β-CD treatment resulted in relatively modest increases of 33.27%, whereas HP-β-CD led to intermediate changes of 83.01% at the same time point. The early and pronounced increase in *b*^⁎^ under α-CD at 6 months, coinciding with its peak chromatic deviation from the control, contrasts with the more gradual and late-onset *b*^⁎^ increases observed under β-CD and HP-β-CD, suggesting that these CD types promote the formation of yellowing-related pigment species at different stages of aging (Jiang et al., 2010).

C* ab and *h*_ab_ (Fig. 1E and Fig. 1F) further highlighted how different CD treatments influenced the temporal evolution of wine color. α-CD-treated wines exhibited the largest ΔC* ab at 3 months (15.36) and maintained elevated values at 6 months (13.92), indicating substantial early- and mid-term alterations in color saturation. β-CD treatments showed minimal ΔC* ab throughout aging, with values as low as 0.75 at 6 months, suggesting relatively minor deviations from the control. HP-β-CD induced intermediate changes, with ΔC* ab ranging from 2.43 to 11.51. For *h*_ab_, α-CD-treated wines displayed pronounced differences, peaking at 87.37 at 6 months, followed by a gradual decrease to 66.02 at 12 months. β-CD-treated wines exhibited smaller and more uniform *h*_ab_, whereas HP-β-CD induced moderate hue differences that were most notable during the mid-term aging period (6–9 months) before declining to 27.24 at 12 months.

Δ*E* integrated the observed chromatic shifts during aging, with higher values indicating greater color differences relative to the control wine (Fig. 1G). Wines treated with α-CD exhibited the largest Δ*E*, peaking at 26.43 at 6 months, whereas β-CD-treated wines showed minimal Δ*E* throughout the storage period (2.00 at 6 months). HP-β-CD treatments were intermediate, with Δ*E* reaching 7.96 at 6 months. Notably, α-CD wines reached peak Δ*E* at 6 months and showed a moderate decline thereafter, whereas β-CD wines reached their minimum Δ*E* at the same time point (2.00) and subsequently increased to 12.00 at 12 months. This inverse temporal relationship indicates that α-CD and β-CD exert their greatest color-altering effects at different stages of the aging process.

*M* and Δ*λ*_max_ were further examined as descriptive UV–Vis spectral parameters to compare treatment- and aging-related changes among wine samples. α-CD treatments produced a pronounced increase in *M*, from 22.17% at 0 months to 108.32% at 6 months, indicating a marked increase in absorbance at 520 nm during early aging (Fig. 1H). β-CD-treated wines exhibited relatively low *M* throughout aging, with a value of 3.81% at 6 months. HP-β-CD induced a moderate increase in *M*, reaching 55.39% at 12 months. Δ*λ*_max_ reflects changes in the absorption maxima of pigments during aging. α-CD-treated wines exhibited a red shift at 3 months (Δ*λ*_max_ = 2.49 nm), followed by blue shifts from 6 to 12 months (−7.56 to −9.91 nm) (Fig. 1I). β-CD treatments showed smaller and relatively stable shifts (5.49 to −4.51 nm), whereas HP-β-CD induced intermediate fluctuations (4.59 to −6.51 nm). These shifts may be related to treatment-dependent changes in wine pigment composition and anthocyanin-derived pigment formation during aging. The transition from positive to negative Δ*λ*_max_ values occurred at 6 months for α-CD and HP-β-CD wines, whereas β-CD wines maintained a positive Δ*λ*_max_ at 6 months before shifting to negative values by 9 months. This difference in transition timing indicates that CD structural properties influence not only the magnitude of spectral change but also the stage of aging at which the dominant pigment structural transformations occur (Colombo et al., 2021).

### 3.2 Dynamic regulation of monomeric phenols by pre-fermentation cyclodextrin addition during wine aging

**Fig. 2:**
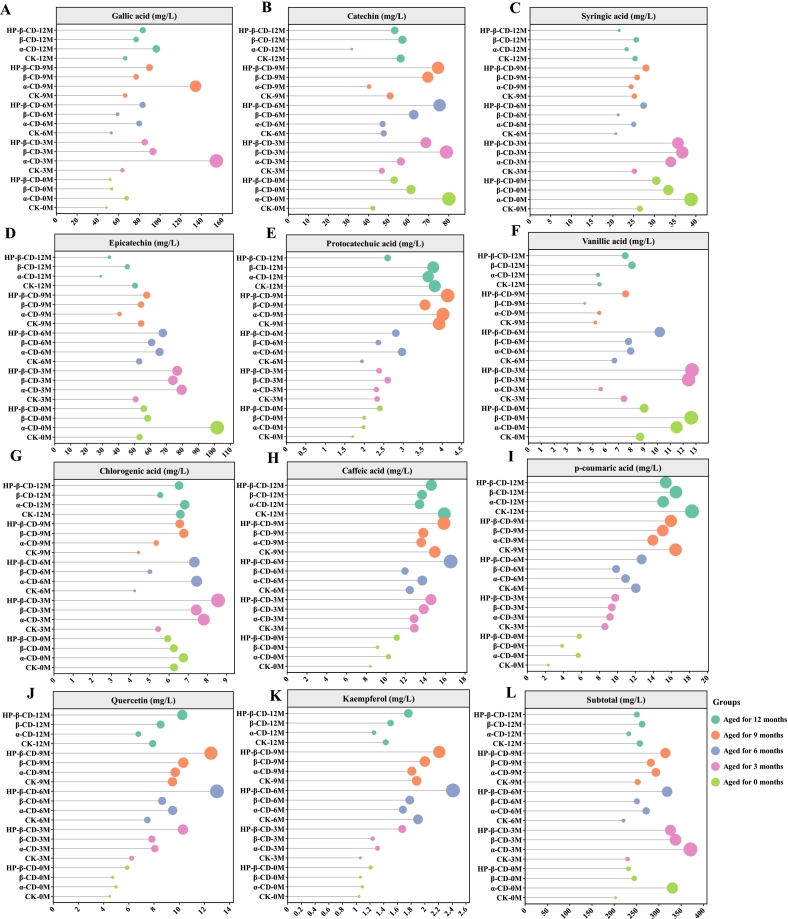
Lollipop plot of monomeric phenols (mg/L) during wine aging. (A) gallic acid; (B) catechin; (C) syringic acid; (D) epicatechin; (E) protocatechuic acid; (F) vanillic acid; (G) chlorogenic acid; (H) caffeic acid; (I) *p*-coumaric acid; (J) quercetin; (K) kaempferol; and (L) subtotal.

Among the non-anthocyanin phenolics monitored in this study, catechin, epicatechin, and gallic acid are particularly relevant to anthocyanin-associated reactions during wine aging. Catechin and epicatechin can serve as nucleophilic partners in direct and acetaldehyde-mediated anthocyanin-flavanol condensation reactions. The stoichiometry and kinetics of these reactions are influenced by the available flavan-3-ol concentration in the wine matrix (Mohammadi et al., 2023). Gallic acid, as a major hydrolysable tannin-derived phenolic in red wine, can engage in supramolecular interactions with anthocyanins, including hydrogen bonding and hydrophobic stacking with the malvidin chromophore. These interactions may affect anthocyanin stability and color expression during aging (Torres-Rochera et al., 2024). Pre-fermentation CD addition markedly influenced monomeric phenol dynamics during aging (Fig. 2). Total monomeric phenols in α-CD-treated wines were consistently higher than the control, reaching approximately 61.20% above control levels at 3 months and remaining around 16.09% higher at 9 months, indicating sustained retention or delayed degradation of monomeric phenols. β-CD and HP-β-CD treatments exhibited intermediate effects, with total monomeric phenols approximately 42–47% higher than the control at 3 months and converging toward control levels by 12 months, reflecting a more moderate and temporally variable modulation of phenolic retention.

Among the individual monomeric phenols, gallic acid, catechin, and epicatechin are discussed in detail because they represent the compound classes identified as mechanistically relevant co-reactants in anthocyanin-associated reactions. Gallic acid in α-CD-treated wines was consistently higher than the control, reaching approximately 2.42-fold higher at 3 months and remaining 49.66% higher at 12 months, indicating sustained retention. By contrast, catechin and epicatechin in α-CD-treated wines were initially higher than the control, increasing by approximately 20.36% and 56.94% at 3 months, respectively, but decreased to about 43% below the control by 12 months. These divergent trajectories suggest that α-CD selectively modulates the wine phenolic matrix in a compound-dependent manner rather than uniformly preserving all monomeric phenols. The sustained elevation of gallic acid indicates that this hydrolysable tannin-derived phenolic may remains available for supramolecular interactions with anthocyanins throughout aging, including hydrogen bonding and hydrophobic stacking with the malvidin chromophore that are known to influence anthocyanin stability and color expression (Torres-Rochera et al., 2024). In contrast, the progressive depletion of catechin and epicatechin below control levels at 12 months suggests their accelerated consumption via condensation reactions; flavan-3-ol concentration is a recognized rate-limiting factor in anthocyanin–flavanol adduct formation (Teng et al., 2021), and this late-stage depletion pattern implies that the balance of co-reactants available for anthocyanin coupling shifts markedly under α-CD treatment over the course of aging. These compound-selective effects likely reflect differences in molecular fit and binding affinity between gallic acid and flavan-3-ols within the α-CD cavity (Fenyvesi et al., 2025).

β-CD wines showed modest increases in gallic acid by 12.54%, catechin by 31.60%, and epicatechin by 14.79% relative to the control at 6 months. HP-β-CD wines displayed larger enhancements, with gallic acid increasing by 56.99%, catechin by 58.72%, and epicatechin by 28.01% compared to the control at 6 months. By 12 months, catechin in β-CD-treated wines remained close to the control, whereas epicatechin was slightly lower. In HP-β-CD wines, both catechin and epicatechin declined below control levels, highlighting CD-type–dependent differences in flavan-3-ol stability during long-term aging. The three CD treatments thus generate distinct co-reactant landscapes that differ not only in absolute compound levels but also in the temporal window during which each substrate remains available for coupling reactions (Cheng et al., 2023). These substrate-level differences provide a phenolic-matrix framework for interpreting the CD-type–dependent variation in anthocyanin transformation profiles presented in the following sections.

The remaining monomeric phenols quantified in this section, including protocatechuic acid, syringic acid, vanillic acid, caffeic acid, quercetin, and kaempferol, are less directly involved in anthocyanin-flavanol condensation reactions. Protocatechuic acid remained largely stable across all treatments, indicating that small and polar phenolics were less affected by CD-mediated retention. Hydroxybenzoic acids, including syringic and vanillic acid, the hydroxycinnamic acid caffeic acid, and flavonols, including quercetin and kaempferol, exhibited transient increases in α-CD-treated wines during early aging. Among them, quercetin showed the most pronounced early rise, whereas β-CD and HP-β-CD induced smaller or more variable effects. These patterns suggest that CD cavity size, substitution pattern, and hydrophobicity may influence the extent to which individual phenolics are retained. Larger or more hydrophobic compounds, such as quercetin and kaempferol, may be more responsive to CD-mediated stabilization, possibly through inclusion complex formation, π-π stacking, or hydrogen bonding. In contrast, smaller and more polar acids were largely unaffected (Liu, Wu, et al., 2025). Although these secondary compounds are not considered primary drivers of anthocyanin condensation reactions, their compound-selective behavior supports the view that CD-mediated phenolic modulation in wine is structurally specific rather than global. Together with the changes in gallic acid and flavan-3-ols, these broader phenolic shifts may alter the co-reactant environment associated with anthocyanin evolution across the three CD treatments. This phenolic-matrix perspective provides useful context for interpreting the anthocyanin evolution and color quality outcomes discussed in the following sections (Huang et al., 2024).

### 3.3 Dynamic regulation of phenolic compounds by pre-fermentation cyclodextrin addition during wine aging

**Fig. 3:**
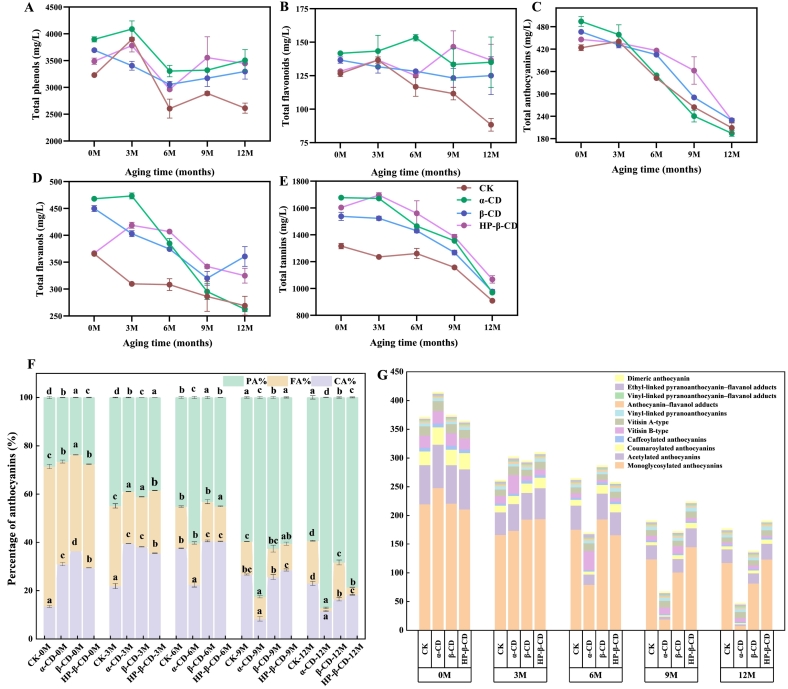
Effects of pre-fermentation addition of α-CD, β-CD, and HP-β-CD on (A) total phenols, (B) total flavonoids, (C) total anthocyanins, (D) total flavanols, (E) total tannins, (F) percentage of anthocyanin forms, and (G) stacked bar chart of anthocyanin subclasses and derivatives during wine aging. Different letters represent significant differences (*p* < 0.05) among different samples within the same aging stage. Note: CA (%), copigmented anthocyanin ratios; FA (%), free anthocyanin ratios; PA (%), polymeric anthocyanin ratios.

Extending beyond their selective effects on individual monomeric phenols, CD additions also influenced the dynamic evolution of the overall phenolic composition during wine aging (Fig. 3). Total phenols fluctuated over time in the control wine, initially increasing at 3 months before declining at 6 months and remaining relatively stable afterwards (Fig. 3A). In contrast, CD-treated wines consistently retained higher total phenol levels throughout aging. At 6 months, α-CD, β-CD, and HP-β-CD wines contained approximately 26.87%, 17.27%, and 13.76% more total phenols than the control, respectively, and this relative advantage persisted through 12 months, with CD treatments maintaining roughly 26–34% higher levels. The consistently higher total phenol levels in CD-treated wines throughout aging suggest that CD addition moderates the net loss of the phenolic matrix, likely through compound-selective stabilization of individual monomeric phenols (Ntuli et al., 2022). Total flavonoids showed pronounced treatment-associated differences. While the control showed a progressive decline, α-CD and HP-β-CD were associated with higher total flavonoid levels throughout aging, reaching approximately 53% higher than the control at 12 months (Fig. 3B). Flavonoids can participate in noncovalent interactions with anthocyanins through π-π stacking and hydrogen bonding, and their sustained retention in CD-treated wines is consistent with the preserved red chromaticity and elevated *M* values observed under α-CD and HP-β-CD throughout aging (Yang et al., 2025).

Total anthocyanins decreased markedly in all wines, with β-CD and HP-β-CD wines retaining higher levels than the control during aging (Fig. 3C). At 6 months, β-CD-treated and HP-β-CD-treated wines retained anthocyanin levels 18.40% and 21.65% higher than the control, respectively, whereas α-CD showed only marginal differences. This result indicates that β-CD and HP-β-CD delayed anthocyanin depletion during mid-term aging, though the differences among treatments diminished by 12 months, suggesting a transient rather than sustained effect on total anthocyanin retention. Total anthocyanin content alone does not account for the divergent color trajectories observed among treatments, as differences in the molecular form of anthocyanins may also contribute to the color outcomes.

Flavanols and tannins exhibited pronounced time-dependent and CD-specific behaviors. α-CD increased total flavanols by 52.85% at 3 months, but this advantage diminished with aging, leading to convergence with the control by 12 months (Fig. 3D). In contrast, β-CD and HP-β-CD sustained higher flavanol contents at later stages, exceeding the control by 34.03% and 20.75%, respectively, at 12 months. The convergence of total flavanols toward control levels specifically under α-CD treatment at 12 months is consistent with the late-stage depletion of individual catechin and epicatechin observed in Section 3.2, suggesting that the aggregate pool of condensation-competent flavan-3-ols was progressively consumed under α-CD treatment during aging. A similar trend was observed for total tannins, which declined steadily in the control but remained higher in β-CD–treated and HP-β-CD–treated wines by 7.47% and 17.49%, respectively, at 12 months (Fig. 3E). Because flavanols and tannins can participate in condensation or association reactions with anthocyanins, their retention provides relevant matrix-level information for interpreting color stability (Verstraeten et al., 2015).

Since wine color depends not only on total anthocyanin concentration but also on the form in which anthocyanins occur, the relative proportions of free, copigmented, and polymeric anthocyanin fractions were analyzed (Fig. 3F). In the control wine, aging was associated with a progressive redistribution of anthocyanin forms, characterized by a decline in free anthocyanins and a concomitant increase in polymeric anthocyanins, whose proportion rose from roughly 28.55% at the beginning of aging to around 59.33% after 12 months. This pattern establishes a baseline trajectory of anthocyanin transformation during aging. CD addition substantially altered this trajectory in a manner that depended on CD type. Among the treatments, α-CD produced the most pronounced redistribution of anthocyanin forms, marked by an early increase in copigmented anthocyanins followed by a strong late-stage dominance of polymeric anthocyanins. At 3 months, copigmented anthocyanins in α-CD-treated wines was markedly higher than in the control, accompanied by a concomitant reduction in free anthocyanins. As aging progressed, polymeric anthocyanins became the predominant fraction in α-CD wines, exceeding 80% of total anthocyanins by 9–12 months, while free anthocyanins declined to marginal levels. This temporal pattern indicates that α-CD promoted an early shift toward copigmented anthocyanins followed by accelerated polymeric pigment formation during late-stage aging, a trajectory that was not observed to the same extent in β-CD or HP-β-CD wines.

In contrast, β-CD and HP-β-CD exerted more moderate and temporally balanced effects on anthocyanin form distribution. In β-CD-treated and HP-β-CD-treated wines, the decline of free anthocyanins followed a trajectory similar to the control, but free anthocyanins were preferentially redistributed toward the copigmented fraction rather than polymeric forms. At 6 months, copigmented anthocyanins remained close to 40% in both β-CD-treated and HP-β-CD-treated wines, while polymeric anthocyanins showed little change. Even at 12 months, polymeric anthocyanin levels in both β-CD-treated and HP-β-CD-treated wines remained substantially lower than in α-CD wines. In β-CD wines, free anthocyanins were largely preserved at levels similar to the control, whereas in HP-β-CD wines, free anthocyanins remained markedly reduced.

Collectively, these results suggest that CDs do not merely preserve total anthocyanins but actively reshape the operationally defined distribution of free, copigmented, and polymeric anthocyanin forms in a structure-dependent manner. The accelerated shift toward polymeric anthocyanins under α-CD coincides with its early and transient increases in total flavanols and its strong retention of gallic acid, as documented in 3.2, 3.3, consistent with a phenolic *co*-reactant environment that favors condensation and polymerization reactions. In contrast, β-CD and HP-β-CD maintained higher proportions of copigmented anthocyanins and preserved free anthocyanins to varying extents, consistent with their more sustained retention of flavanols and tannins. This redistribution pattern aligns with the more moderate and late-onset chromatic deviations observed under these treatments in Fig. 1.

### 3.4 Dynamic regulation of anthocyanin and their derivatives by pre-fermentation cyclodextrin addition during wine aging

**Fig. 4:**
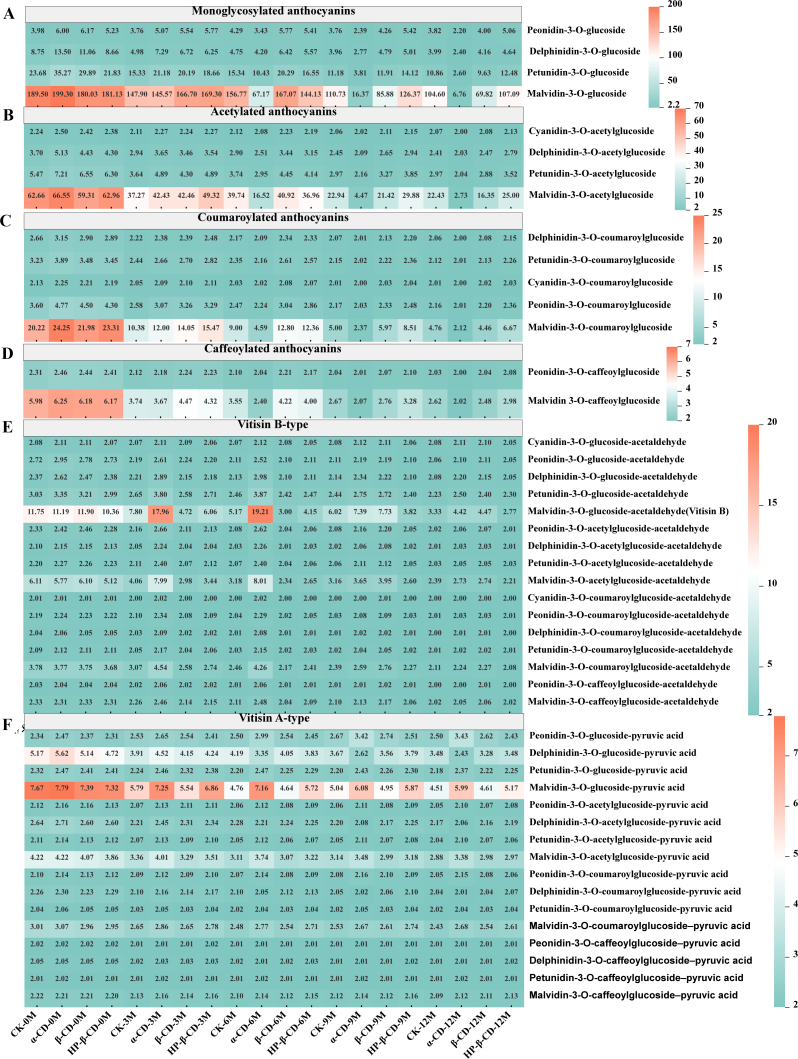

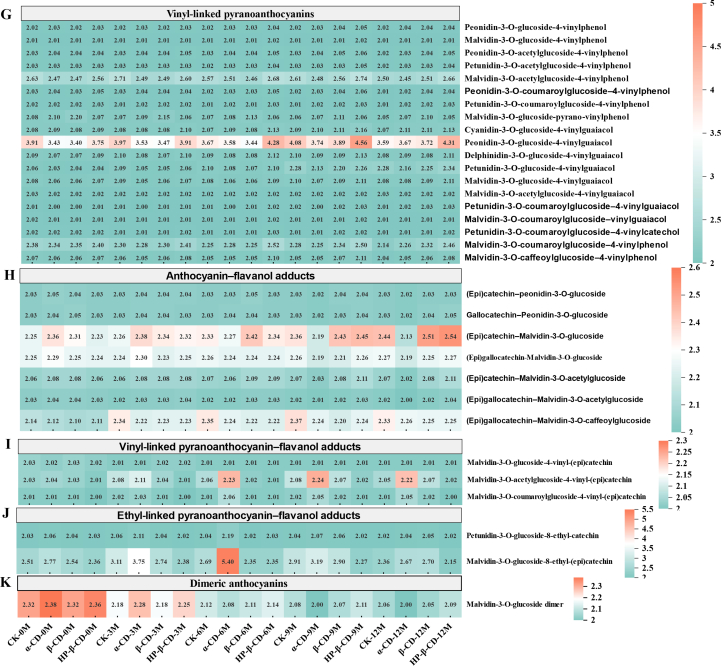
Heatmap of anthocyanins and their derivatives in wine during wine aging. (A) monoglycosylated anthocyanins; (B) acetylated anthocyanins; (C) coumaroylated anthocyanins; (D) caffeoylated anthocyanins; (E) vitisin B-type anthocyanins; (F) vitisin A-type anthocyanins; (G) vinyl-linked pyranoanthocyanins; (H) anthocyanin–flavanol adducts; (I) vinyl-linked pyranoanthocyanin–flavanol adducts; (J) ethyl-linked pyranoanthocyanin–flavanol adducts; and (K) dimeric anthocyanins. Red and blue colors represent higher and lower contents, respectively, and the numerical values displayed correspond to the concentrations of anthocyanins and their derivatives. (For interpretation of the references to color in this figure legend, the reader is referred to the web version of this article.)

To further examine which anthocyanin groups contributed to the changes observed in total anthocyanins and anthocyanin fractions, individual anthocyanins and derived pigments were analyzed across 11 structural categories (Fig. 3G, Fig. 4, and Table 1). Monoglycosylated anthocyanins (M-GA) are the main contributors to wine color and its evolution during aging, making their stability and transformation particularly critical for understanding long-term chromatic development (Fig. 4A). Among these, malvidin-3-*O*-glucoside was the most abundant, displaying pronounced aging-related depletion with kinetics that varied markedly depending on CD structure. In the control wine, malvidin-3-*O*-glucoside declined by 44.80% over 12 months, reflecting progressive degradation and transformation during aging. This loss was strongly accelerated by α-CD, with levels decreasing by 96.61%, whereas reductions were substantially smaller under β-CD (61.22%) and HP-β-CD (40.88%). Similar trends were observed for other major M-GA, including petunidin- and delphinidin-based derivatives, highlighting the generalized effect of α-CD in promoting early anthocyanin consumption.

**Table 1:** Effects of pre-fermentation addition of α-CD, β-CD, and HP-β-CD on classified subclasses of anthocyanins and their derivatives (mg/L) during wine aging (M = months).

		CK	α-CD	β-CD	HP-β-CD
M-GA	0 M	219.91 ± 6.11a	248.07 ± 7.35b	221.15 ± 10.59a	210.85 ± 6.19a
	3 M	165.97 ± 6.20a	173.10 ± 4.79b	193.15 ± 5.64a	193.97 ± 11.39a
	6 M	175.14 ± 5.11b	79.24 ± 4.32a	193.54 ± 5.32c	165.66 ± 8.66b
	9 M	123.63 ± 6.16c	19.34 ± 2.47a	100.84 ± 4.40b	144.92 ± 6.97d
	12 M	117.26 ± 3.27c	7.96 ± 2.23a	81.61 ± 7.32b	123.28 ± 10.72c
AcA	0 M	68.08 ± 4.81a	75.40 ± 3.16b	66.71 ± 3.74a	69.94 ± 3.98a
	3 M	39.96 ± 2.26a	47.24 ± 3.16b	46.46 ± 2.85b	54.02 ± 5.43c
	6 M	42.50 ± 2.85bc	18.06 ± 2.36a	45.04 ± 3.56c	40.43 ± 3.65b
	9 M	24.41 ± 2.67b	4.73 ± 2.11a	23.44 ± 2.54b	32.83 ± 2.50c
	12 M	23.88 ± 2.12c	2.81 ± 2.02a	17.79 ± 2.39b	27.43 ± 3.72d
CmA	0 M	23.85 ± 1.18a	30.32 ± 1.77b	27.06 ± 2.13ab	28.13 ± 2.13ab
	3 M	11.68 ± 0.55a	14.20 ± 0.90b	16.50 ± 0.52c	18.17 ± 1.01c
	6 M	10.02 ± 0.56b	5.10 ± 0.16a	14.87 ± 0.48c	14.19 ± 0.96c
	9 M	5.41 ± 0.15b	2.43 ± 0.03a	6.67 ± 0.31c	9.59 ± 0.29d
	12 M	5.11 ± 0.19b	2.14 ± 0.00a	4.90 ± 0.18b	7.47 ± 0.45c
CfA	0 M	6.29 ± 0.28a	6.71 ± 0.31a	6.62 ± 0.42a	6.58 ± 0.16a
	3 M	3.86 ± 0.12a	3.85 ± 0.14a	4.71 ± 0.16b	4.55 ± 0.09b
	6 M	3.66 ± 0.08b	2.44 ± 0.03a	4.43 ± 0.10c	4.17 ± 0.18c
	9 M	2.71 ± 0.06b	2.08 ± 0.01a	2.83 ± 0.03c	3.38 ± 0.08d
	12 M	2.65 ± 0.02c	2.02 ± 0.00a	2.52 ± 0.02b	3.06 ± 0.07d
VitB	0 M	21.16 ± 1.06b	21.39 ± 1.23b	21.97 ± 1.13b	18.71 ± 0.58a
	3 M	12.85 ± 1.32c	32.31 ± 1.97d	7.88 ± 0.06a	10.00 ± 0.14b
	6 M	7.96 ± 0.07c	33.32 ± 0.57d	4.39 ± 0.03a	6.29 ± 0.17b
	9 M	8.67 ± 0.28b	11.65 ± 0.06c	12.44 ± 0.31d	5.58 ± 0.10a
	12 M	4.36 ± 0.08b	6.54 ± 0.11c	6.52 ± 0.06c	3.61 ± 0.05a
VitA	0 M	16.31 ± 1.29b	17.45 ± 1.43c	15.93 ± 1.60ab	15.15 ± 1.37a
	3 M	11.24 ± 0.17a	15.01 ± 0.40c	11.44 ± 0.24a	13.27 ± 0.43b
	6 M	9.97 ± 0.18a	13.36 ± 0.21c	9.88 ± 0.30a	11.10 ± 0.13b
	9 M	9.86 ± 0.21a	11.41 ± 0.13b	9.79 ± 0.16a	11.25 ± 0.26b
	12 M	8.46 ± 0.04a	10.91 ± 0.12d	8.86 ± 0.11b	9.56 ± 0.21c
VPyA	0 M	5.62 ± 0.11b	4.86 ± 0.15a	4.94 ± 0.16a	5.40 ± 0.16b
	3 M	5.67 ± 0.22b	4.91 ± 0.16a	4.93 ± 0.17a	5.63 ± 0.21b
	6 M	5.13 ± 0.21a	5.03 ± 0.10a	4.83 ± 0.20a	6.37 ± 0.27b
	9 M	5.84 ± 0.20b	5.19 ± 0.19a	5.73 ± 0.21b	6.99 ± 0.30c
	12 M	5.00 ± 0.23a	5.14 ± 0.16ab	5.48 ± 0.12b	6.55 ± 0.17c
A–F adducts	0 M	2.79 ± 0.01ab	2.99 ± 0.03c	2.86 ± 0.06b	2.72 ± 0.03a
	3 M	2.98 ± 0.02a	3.10 ± 0.04b	2.99 ± 0.03a	2.99 ± 0.01a
	6 M	3.09 ± 0.01c	2.88 ± 0.02a	3.10 ± 0.03c	2.98 ± 0.02b
	9 M	3.15 ± 0.03c	2.70 ± 0.02a	3.01 ± 0.01b	3.17 ± 0.01c
	12 M	3.19 ± 0.01b	2.64 ± 0.03a	3.18 ± 0.04b	3.29 ± 0.03c
VPyA–F	0 M	2.07 ± 0.00b	2.06 ± 0.00b	2.06 ± 0.00b	2.03 ± 0.00a
	3 M	2.10 ± 0.00c	2.15 ± 0.01d	2.06 ± 0.00b	2.03 ± 0.00a
	6 M	2.08 ± 0.00b	2.30 ± 0.02c	2.04 ± 0.00a	2.03 ± 0.00a
	9 M	2.11 ± 0.01b	2.30 ± 0.01c	2.10 ± 0.00b	2.04 ± 0.00a
	12 M	2.06 ± 0.00b	2.28 ± 0.02d	2.11 ± 0.01c	2.03 ± 0.00a
EPyA–F	0 M	2.53 ± 0.00b	2.83 ± 0.04c	2.57 ± 0.02b	2.39 ± 0.02a
	3 M	3.17 ± 0.01c	3.85 ± 0.13d	2.78 ± 0.04b	2.41 ± 0.03a
	6 M	2.72 ± 0.02b	5.59 ± 0.17c	2.37 ± 0.02a	2.37 ± 0.01a
	9 M	2.95 ± 0.02b	3.26 ± 0.04c	2.96 ± 0.06b	2.29 ± 0.01a
	12 M	2.38 ± 0.01b	2.70 ± 0.01c	2.75 ± 0.02d	2.16 ± 0.01a
DiA	0 M	2.32 ± 0.01a	2.38 ± 0.01b	2.32 ± 0.03a	2.36 ± 0.01b
	3 M	2.18 ± 0.00a	2.28 ± 0.00c	2.18 ± 0.01a	2.25 ± 0.02b
	6 M	2.12 ± 0.00c	2.08 ± 0.00a	2.11 ± 0.01b	2.14 ± 0.02d
	9 M	2.08 ± 0.01b	2.00 ± 0.00a	2.07 ± 0.00b	2.11 ± 0.01c
	12 M	2.06 ± 0.00b	2.00 ± 0.00a	2.05 ± 0.00b	2.09 ± 0.01c

Note: Monoglycosylated anthocyanins (M-GA), acetylated anthocyanins (AcA), coumaroylated anthocyanins (CmA), caffeoylated anthocyanins (CfA), Vitisin B-type anthocyanins (VitB), Vitisin A-type anthocyanins (VitA), vinyl-linked pyranoanthocyanins (VPyA), anthocyanin–flavanol adducts (A–F adducts), vinyl-linked pyranoanthocyanin–flavanol adducts (VPyA–F), ethyl-linked pyranoanthocyanin–flavanol adducts (EPyA–F), and dimeric anthocyanins (DiA).

Average and standard deviation (x ± SD) were calculated for three samples.

Different letters in the same row represent significant differences (*p* < 0.05) among samples within the same aging stage.

In control wines, the total M-GA decreased by approximately 46.68% from 0 to 12 months. α-CD markedly accelerated this depletion, with levels falling by over 96.79%, consistent with the rapid conversion from free to polymeric anthocyanins and the strong late-stage polymeric anthocyanins accumulation described above. By contrast, β-CD and HP-β-CD wines retained higher proportions of M-GA, with reductions of 63.10% and 41.54%, respectively. These results indicate that the larger or more flexible CD cavities may stabilize monomeric anthocyanins by partially shielding reactive sites, thereby limiting their participation in condensation or oxidative transformations (Su et al., 2024).

Acylated anthocyanins (AcA), including acetylated, coumaroylated, and caffeoylated forms, represent a structurally distinct subclass with increased hydrophobicity and steric bulk, making their stability particularly sensitive to microenvironmental factors. In α-CD–treated wines, acetylated and coumaroylated malvidin derivatives exhibited transient early enrichment, with malvidin-3-*O*-acetylglucoside and malvidin-3-*O*-coumaroylglucoside increasing by 13.86% and 15.62% at 3 months, respectively, relative to the control wine (Fig. 4B). However, these compounds declined sharply thereafter, reaching 8.22-fold and 2.24-fold decreases over 12 months, consistent with accelerated mid-stage to late-stage degradation and transformation. In contrast, HP-β-CD–treated wines displayed substantially higher retention of AcA, with malvidin-3-*O*-acetylglucoside and malvidin-3-*O*-coumaroylglucoside decreasing only 11.44% and 40.12% over 12 months compared with the control wine, which corresponds to approximately 9.16-fold and 3.14-fold higher levels than those in α-CD wines. These patterns suggest that the larger and more flexible cavity of HP-β-CD may preferentially accommodate the hydrophobic and sterically accessible portions of AcA, limiting their exposure to nucleophilic or oxidative agents and thereby slowing degradation. Such selective stabilization could also modulate the kinetics of condensation and anthocyanin-derived pigment formation, contributing to the prolonged preservation of AcA and sustained chromaticity in HP-β-CD–treated wine (Musuc, 2024).

Vitisin-type pyranoanthocyanins, including both A-types and B-types, are important derived pigments that contribute to the long-term color stability of wine, representing stable end-products of anthocyanin condensation and modification pathways. Among individual compounds, vitisin B (malvidin-3-*O*-glucoside–acetaldehyde) was particularly sensitive to CD-mediated modulation. In the control wine, vitisin B decreased by 71.63% over 12 months, indicating that its formation did not compensate for concurrent degradation or further conversion. In α-CD–treated wines, vitisin B rapidly accumulated, increasing by 60.50% at 3 months relative to the initial level and peaking at 70.67% above the control at 6 months, before slightly declining at 9 months. This early enrichment is likely due to the small, rigid α-CD cavity, which partially confines the anthocyanin molecule, promoting close-range interactions with acetaldehyde while keeping the reactive C4/C5 site accessible (Qiao et al., 2025). By contrast, β-CD initially suppressed vitisin B formation due to partial shielding of key reactive sites, but delayed release or reorientation of the anthocyanin within the cavity allowed formation to exceed the control at 9 and 12 months (+28.43% and + 34.15%, respectively). In HP-β-CD wines, vitisin B remained consistently below the control throughout aging, suggesting that the larger, flexible cavity effectively shields reactive anthocyanin sites, limiting encounters with acetaldehyde and suppressing pyranoanthocyanin formation (Falsafi et al., 2023).

Considering total Vitisin-type pyranoanthocyanins, similar trends were observed. In α-CD wines, both B-types and A-types were markedly elevated from early aging, reaching approximately 4.18-fold and 1.34-fold of the control at 6 months, and remaining 50.01% and 28.95% higher at 12 months. In β-CD wines, total vitisin levels were initially lower than the control but gradually increased during aging. By 12 months, total B-type and A-type vitisins reached 49.53% and 4.70% of the control, respectively, reflecting a moderate accumulation. In contrast, HP-β-CD consistently induced only limited enrichment throughout aging. Overall, these observations indicate that CD structure modulates vitisin-type pyranoanthocyanin formation by controlling the balance between molecular encapsulation, spatial proximity, and accessibility of reactive anthocyanin sites (Sendri & Bhandari, 2024). Small, rigid α-CD favors early condensation with acetaldehyde, leading to rapid vitisin accumulation, whereas β-CD and HP-β-CD stabilize anthocyanins in less reactive conformations, slowing pyranoanthocyanin formation and producing distinct aging kinetics.

Although vinyl-linked pyranoanthocyanins (VPyA) remained relatively minor components in wines, they play an important role in the long-term color stability and evolution of aged wines. Under β-CD, VPyA exhibited modest enrichment during early aging, consistent with the slower depletion of free anthocyanins in this treatment. By contrast, α-CD generally suppressed their formation, particularly during late aging, whereas HP-β-CD promoted gradual accumulation at later stages, suggesting that its larger and more flexible cavity stabilizes reactive intermediates and facilitates their progressive formation. More complex anthocyanin-flavanol adducts (A-F adducts), such as (*epi*)catechin-malvidin-3-*O*-glucoside, were relatively scarce but displayed pronounced treatment-dependent differences. Their accumulation was highest under α-CD, consistent with the progressive consumption of catechin and epicatechin as nucleophilic condensation partners, shifting anthocyanin transformation pathways toward irreversible cross-linking products. Under β-CD and HP-β-CD, levels of these adducts remained largely comparable to the control, consistent with the preservation of free anthocyanins and flavanols and the slower progression toward complex polymeric structures. Similarly, vinyl- and ethyl-linked pyranoanthocyanin–flavanol adducts were most abundant in α-CD–treated wines, whereas levels under β-CD and HP-β-CD remained largely comparable to the control. Dimeric anthocyanins were generally stable across all treatments, showing only modest increases under α-CD, indicating limited susceptibility to CD-mediated modulation.

Taken together, these results indicate that CDs modulate anthocyanin aging not merely by preserving total pigment levels but by redistributing flux among competing transformation pathways in a structure-dependent manner. Mechanistically, α-CD appears to function as an active kinetic regulator rather than a passive stabilizer: rather than keeping free anthocyanins intact, it was associated with accelerated conversion of unstable monomeric anthocyanins into vitisin-type pyranoanthocyanin networks (particularly vitisin B), channeling monomer depletion toward chromatic products that are more resistant to hydration-induced bleaching. This may underlie the early enhancement of red intensity (*a*^⁎^ and C* ab) observed in α-CD-treated wines, even as monomeric anthocyanin concentrations declined. By contrast, β-CD and HP-β-CD were associated with better retention of monomeric and acylated anthocyanins, suggesting a passive stabilizing role that slowed polymeric pigment accumulation and sustained long-term color stability.

### 3.5 Principal component analysis of individual anthocyanins and their derivatives during wine aging

**Fig. 5:**
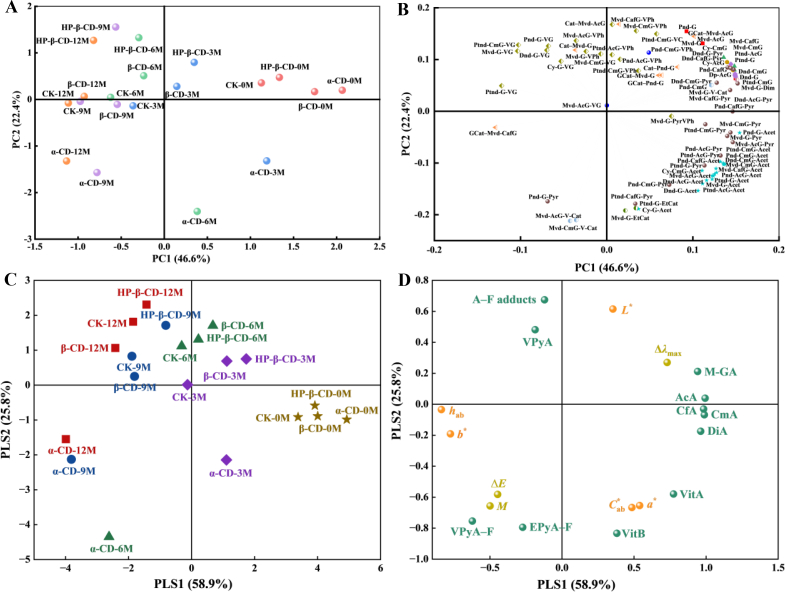
Principal component analysis (PCA) score plot (A), loading plot of anthocyanins and their derivatives (B), partial least squares regression (PLSR) score plot (C), and PLSR loading plot (D) of subclasses of anthocyanin and their derivatives, CIELAB color parameters, and UV–Vis spectral parameters during wine aging. Note: Monoglycosylated anthocyanins (M-GA), acetylated anthocyanins (AcA), coumaroylated anthocyanins (CmA), caffeoylated anthocyanins (CfA), Vitisin B-type anthocyanins (VitB), Vitisin A-type anthocyanins (VitA), vinyl-linked pyranoanthocyanins (VPyA), anthocyanin–flavanol adducts (A–F adducts), vinyl-linked pyranoanthocyanin–flavanol adducts (VPyA–F), ethyl-linked pyranoanthocyanin–flavanol adducts (EPyA–F), and dimeric anthocyanins (DiA). Detailed abbreviations and full names of individual anthocyanins and their derivatives are listed in Table S1. The same abbreviations are used in the figures below as figure captions for consistency.

PCA was employed to integrate the distribution patterns of individual anthocyanins and their derivatives across CD treatments and aging stages, enabling visualization of global chemical differentiation associated with pre-fermentation CD addition (Fig. 5A and B). The first two principal components accounted for 46.6% and 22.4% of the total variance, respectively, capturing both aging-driven evolution and treatment-specific modulation of anthocyanin transformation pathways. PC1 primarily reflected the progression of wine aging and the redistribution of anthocyanins from monomeric to derived forms. Most M-GA and AcA exhibited positive loadings on PC1, whereas vinyl-linked derivatives, and several A–F adducts showed negative or weakly positive contributions. Consistent with this loading plot, score plot of all wines shifted progressively toward negative PC1 values with aging, indicating a general depletion of monomeric anthocyanins accompanied by the gradual emergence of structurally derived pigments.

Notably, α-CD–treated wines displayed a more pronounced and earlier displacement along PC1 compared with the control and β-CD–based treatments. At 3 and 6 months, α-CD wines already occupied regions of the score plot typically associated with later aging stages, suggesting an accelerated dynamic evolution of the anthocyanins. This shift aligns with the loading pattern in which polymeric and pyranoanthocyanin-related anthocyanins dominate the negative side of PC1, supporting the interpretation that α-CD promotes rapid consumption of monomeric precursors and their diversion toward irreversible condensation pathways. By contrast, β-CD and HP-β-CD wines showed a more gradual movement along PC1, remaining closer to the origin during early aging and overlapping more closely with the control at intermediate stages.

PC2 provided additional discrimination related to anthocyanin structural classes and modes of derivatization. Several acetylated anthocyanins, certain pyranoanthocyanins, and vinyl- or ethyl-linked anthocyanin–flavanol adducts exhibited substantial loadings on PC2, indicating that this axis reflects differences in substitution patterns, steric complexity, and cross-linking behavior rather than simple abundance. HP-β-CD wines were consistently displaced toward higher PC2 scores, particularly at 6–12 months, whereas α-CD wines tended toward lower PC2 values. This divergence suggests that HP-β-CD may preferentially stabilize or allow the gradual accumulation of structurally complex but less extensively cross-linked derivatives, whereas α-CD appears associated with products linked to rapid, early-stage condensation and cross-linking. Collectively, the PCA score trajectories demonstrate that CD structure modulates anthocyanin evolution not only by shifting overall transformation extent but also by reorienting chemical trajectories within multivariate space, producing treatment-specific differentiation that persists across all aging stages (Ali et al., 2024).

### 3.6 PLSR of anthocyanin subclasses and their derived pigments in relation to CIELAB color and UV–vis spectral parameters during wine aging

PLSR was applied to examine the relationships between anthocyanin subclasses, derived pigments, and color-related parameters during wine aging under different CD treatments. The first latent variable (PLS1) primarily reflected monomeric anthocyanin abundance, with freshly fermented wines showing high positive scores driven by M-GA and AcA. During aging, PLS1 shifted progressively toward negative values, corresponding to monomer decline and accumulation of derived pigments. α-CD wines exhibited the fastest and most pronounced shift, accompanied by accelerated decreases in *L*^⁎^ and simultaneous increases in Δ*E* and *M*, consistent with enhanced formation of polymeric pigments and pyranoanthocyanin–flavanol adducts. In contrast, β-CD and HP-β-CD wines followed slower, more moderate PLS1 trajectories, aligned with more gradual *L*^⁎^ decreases and delayed accumulation of derived pigments, suggesting that larger or more flexible CD cavities stabilize reactive intermediates and moderate condensation kinetics.

PLS2 captured differences in the mode of anthocyanin transformation. α-CD wines displayed early negative PLS2 scores, corresponding to strong loadings of vitisin-type pyranoanthocyanins and higher *a*^⁎^ and C* ab, consistent with early retention of red chroma via rapid pyranoanthocyanin formation. In contrast, HP-β-CD wines gradually shifted toward positive PLS2, associated with VPyA and A–F adducts, reflecting slower but sustained formation of complex derivatives, likely due to partial stabilization of reactive intermediates within the flexible HP-β-CD cavity. β-CD wines exhibited intermediate behavior. The alignment of *a*^⁎^ and C* ab with polymeric and pyranoanthocyanin-associated loadings reinforces the coupling between chemical transformation pathways and macroscopic chromatic evolution, while the opposing orientations of M-GA and polymeric derivatives in the loading plot support a competitive relationship between preservation and transformation pathways during aging.

**Fig. 6:**
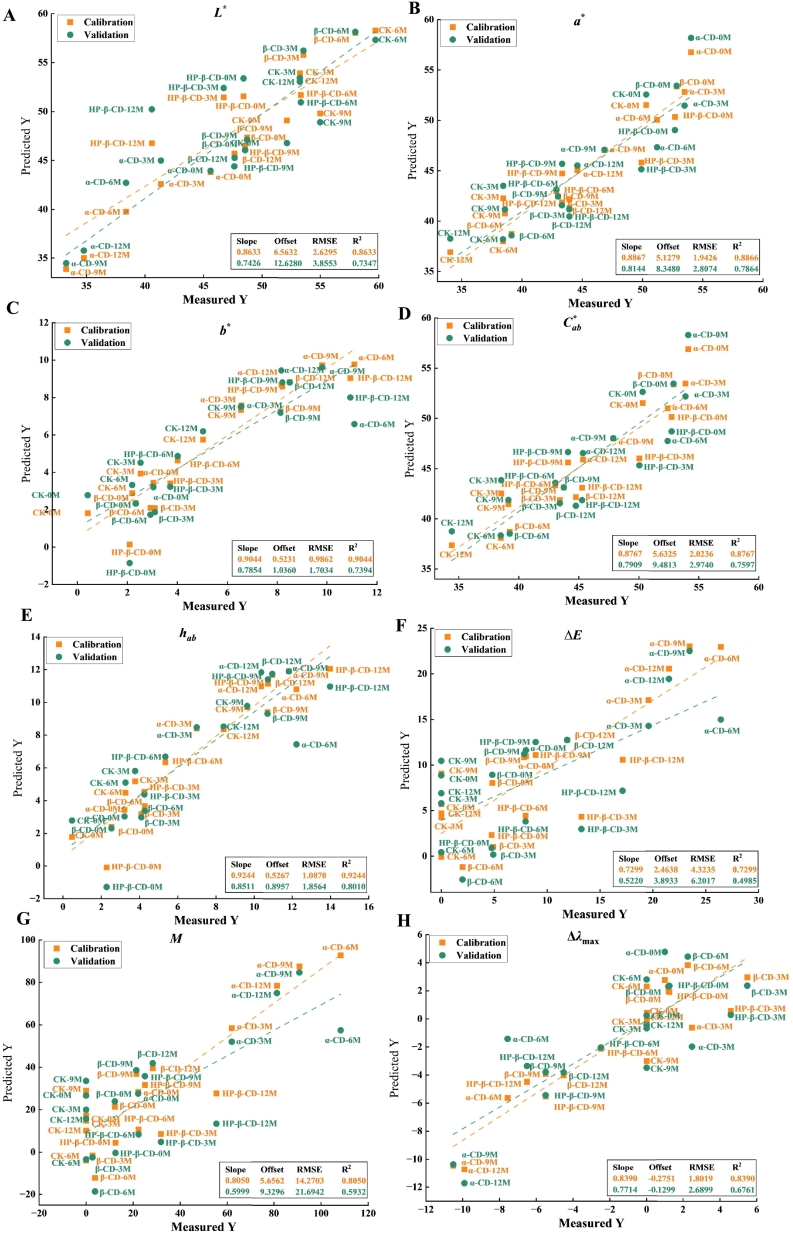
Partial least squares regression (PLSR) analysis of anthocyanins and their derivatives in relation to CIELAB color parameters and UV–Vis spectral parameters: (A) *L*^⁎^ (lightness), (B) *a*^⁎^ (red-green tone), (C) *b*^⁎^ (yellow-blue tone), (D) C* ab (chroma), (E) *h*_ab_ (hue angle), (F) color differences (Δ*E*), (G) hyperchromic effect (*M*), and (H) bathochromic shift (**Δ***λ*_max_) during wine aging. (For interpretation of the references to color in this figure legend, the reader is referred to the web version of this article.)

To quantitatively characterize these relationships, PLSR models were developed to link anthocyanin subclasses and their derivatives with colorimetric parameters during wine aging under different CD treatments (Fig. 6). For the CIELAB color parameters, models showed good calibration and predictive performance, with R^2^_cal_ ranging from 0.863 to 0.924 and R^2^_val_ from 0.735 to 0.801, and corresponding RMSEC and RMSECV values of 0.99–2.63 and 1.70–3.86, respectively. The *M* and Δ*λ*_max_ yielded acceptable calibration (R^2^_cal_ = 0.805 and 0.839, respectively) but lower cross-validation performance (R^2^_val_ = 0.593 and 0.676, respectively), reflecting greater variability in UV–Vis spectral responses across treatments. Δ*E* showed the weakest predictive validity (R^2^_cal_ = 0.730, R^2^_val_ = 0.499), consistent with the nonlinear dynamics of total color deviation that are not fully captured by the linear PLSR framework, as also reflected in the scatter at extreme values. *L*^⁎^ decreased progressively during aging in both α-CD and HP-β-CD wines, with α-CD wines exhibiting the most rapid decline, particularly during early to mid-aging stages. These observations are consistent with PLS1 analysis and the lightness differences shown in Fig. 1B, confirming the trends across different CD treatments. Analysis of the *L*^⁎^ scatter plot indicates that the slope and RMSE are slightly less accurate at extreme low values, suggesting that late-stage formation of high-molecular-weight derivatives may involve nonlinear dynamics not fully captured by the linear PLSR model.

*a*^⁎^ and C* ab decreased progressively across all treatments, with treatment-specific modulation of red hue intensity. α-CD wines exhibited higher predicted a values at both the initial stage (0 M) and the late aging stage (9–12 M), whereas intermediate aging (3–6 M) showed relatively lower *a*^⁎^ values. This pattern may reflect transient redistribution of red pigments, potentially associated with early formation and late stabilization of vitisin-type pyranoanthocyanins. In contrast, HP-β-CD wines displayed a more gradual decline in *a*^⁎^, consistent with distributed formation of complex pigment derivatives throughout aging. *b*^⁎^ exhibited notable variability during both early (0–3 M) and late aging (12 M) in CK and HP-β-CD wines, with predicted values deviating from measured ones. This instability may reflect transient pigment rearrangements or delayed cyclization of reactive intermediates.

UV–Vis spectral parameters (*M* and Δ*λ*_max_), together with the overall color difference Δ*E*, provide complementary information on treatment- and aging-related color evolution. Δ*E* and *M* increased most rapidly in α-CD wines, corresponding to enhanced condensation and pyranoanthocyanin formation, while HP-β-CD wines exhibited slower but sustained accumulation. The *M* scatter plots revealed treatment-specific patterns during aging. In α-CD and HP-β-CD wines, predicted *M* values underestimated measured values at later stages, whereas in β-CD wines, predictions initially underestimated early-stage *M* but exceeded measured values at later aging. This shift in β-CD wines may reflect delayed condensation in early aging followed by more extensive polymerization or secondary pathways in late aging, suggesting that CD cavity size and flexibility modulate the kinetics and mechanisms of pigment polymerization. Δ*λ*_max_ showed larger deviations between predicted and measured values during early aging (0–6 M), but predictions aligned closely with measurements in late stages (9–12 M). This pattern likely reflects rapid, heterogeneous pigment transformations during early aging, including condensation and cyclization, whereas pigment equilibria stabilize during later aging, leading to more consistent absorption maxima (Alvarez et al., 2014). In summary, anthocyanin subclasses and their derivatives closely govern wine color evolution, with *L*^⁎^, *a*^⁎^, and *C*^*⁎*^_*ab*_ reflecting monomer decline and polymer/pyranoanthocyanin formation, while Δ*E*, *M*, and Δ*λ*_max_ capture transient and late-stage transformations. The PLSR analysis complements the univariate results by demonstrating that treatment-dependent differences in color parameters were closely coupled to coordinated shifts in anthocyanin subclass composition and derived pigment accumulation during aging.

### 3.7 Mantel and canonical correspondence analysis of anthocyanin subclasses and their derived pigments, CIELAB color parameters and UV–vis spectral changes during wine aging

**Fig. 7:**
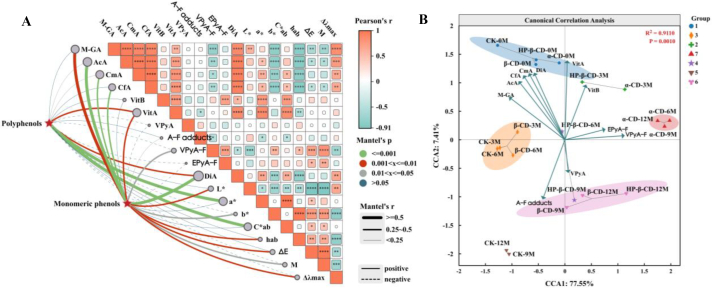
Mantel correlation analysis among anthocyanins and their derivatives, CIELAB color parameters, UV–Vis spectral parameters, monomeric phenols, and polyphenols (A), and canonical correspondence analysis (CCA) of anthocyanins and their derivatives with CLELAB color parameters and UV–Vis spectral parameters (B). The angles between vectors and the lines connecting the origin to sample points indicate the direction and strength of correlations: acute angles denote positive correlations (smaller angles indicate stronger correlations), obtuse angles denote negative correlations, and right angles indicate weak or negligible correlations.

Mantel correlation analysis and CCA were applied to map structural relationships between anthocyanin subclasses and color parameters across treatments and aging stages (Fig. 7A and 7B). Mantel correlation analysis revealed structured relationships between anthocyanin subclasses and their derivatives and both CIELAB color parameters and UV–Vis spectral parameters. Monomeric anthocyanins, including monoglycosylated, acetylated, coumaroylated, and caffeoylated forms, were positively associated with red intensity, with monoglycosylated anthocyanins showing the strongest correlations with *a*^⁎^ and C* ab, suggesting that early-stage red hue is predominantly determined by monomer abundance. Notably, *L*^⁎^ exhibited consistent negative correlations with high-molecular-weight derivatives, except for A–F adducts, indicating that the accumulation of high-molecular-weight pigment structures is generally associated with progressive darkening during aging. In parallel, *b*^⁎^ and *h*_ab_ exhibited strong positive associations with VPyA, A–F adducts, vinyl-linked pyranoanthocyanin–flavanol adducts (VPyA–F), and ethyl-linked pyranoanthocyanin–flavanol adducts (EPyA–F), with the strongest correlations observed for VPyA–F, reaching 0.58 for *b*^⁎^ and 0.50 for *h*_ab_. These patterns suggest that the development of pyranoanthocyanin–flavanol networks may enhance yellow tonal contributions and promote systematic hue shifts toward the yellow–orange domain, potentially arising from polymerization-driven expansion of conjugated systems and associated changes in electronic structure during wine aging (He et al., 2010).

In contrast, *M* and Δ*E* showed significant positive correlations with late-stage pyranoanthocyanin and polymeric derivatives, particularly Vitisin B, VPyA–F, and EPyA–F. Among these, VPyA–F exhibited the strongest associations, with correlation coefficients of 0.80 for *M* and 0.71 for Δ*E*. This pattern suggests that the accumulation of polymeric and ring-closed pyranoanthocyanin–flavanol structures increasingly contributes to UV–Vis spectral changes and color evolution during aging. The extended conjugation and steric shielding associated with these derivatives may contribute to enhanced pigment stability and reduced bleaching, thereby sustaining macroscopic color stability and underscoring the importance of late-stage polymerization in the optical evolution of aging wine (Solano et al., 2006). Overall, the Mantel correlations support the interpretation that wine color evolution during aging was associated with both monomeric anthocyanins and derived pigments. Early-stage visual properties are largely dictated by monomer abundance, whereas late-stage brightness and chromatic divergence are increasingly governed by high-molecular-weight derivatives. CD interventions appear to fine-tune the timing and extent of competing chemical pathways, linking molecular pigment dynamics to macroscopic color evolution in a treatment-specific manner.

CCA revealed distinct trajectories of anthocyanin evolution under CD treatments. Along CCA1, monomeric anthocyanins, including monoglycosylated, acylated, and dimeric forms, occupied the negative axis, reflecting early-stage wines such as CK-0 M and HP-β-CD-0 M, whereas pyranoanthocyanin–flavanol derivatives, particularly vinyl- and ethyl-linked adducts, aligned with the positive axis, notably in α-CD-treated samples at 3–12 months. *M* and Δ*E* were closely associated with the positive region, consistent with the increasing contribution of VPyA–F and related derivatives to color stabilization during later aging. Control wines remained on the negative side throughout aging, reflecting sustained monomer dominance. In contrast, α-CD–treated wines shifted rapidly to the positive side, favoring condensation and pyranoanthocyanin–flavanol polymerization, likely by reducing solvent accessibility or concentrating reactive intermediates. β-CD induced a more moderate progression along CCA1, and HP-β-CD exhibited intermediate behavior, initially resembling monomer-rich regions before gradually moving toward polymer-associated space. These patterns suggest that structural differences among CD types drive divergent pigment transformation trajectories during wine aging.

CCA2 primarily reflected variations associated with structural modification of pigment rather than molecular size. Vitisin B-type anthocyanins (VitB) and vitisin A-type anthocyanins (VitA) loaded strongly on the positive side, suggesting their contribution to chromatic continuity during intermediate aging. In contrast, A–F adducts and VPyA were oriented toward negative values, implying that polymerization and ring closure may progressively modulate the yellow–red balance. Notably, α-CD-treated wines maintained elevated CCA2 scores throughout aging, whereas β-CD and HP-β-CD wines gradually shifted downward, indicating more extensive formation of polymeric adducts. This vertical redistribution suggests that aging involves partial replacement or overshadowing of pyranoanthocyanins by complex adducts rather than a simple linear accumulation, with CD cavity properties potentially influencing the relative prominence and timing of these pathways. Together with the preceding analyses, the multivariate results support a data-driven link between anthocyanin subclass composition, derived pigment accumulation, and macroscopic color evolution, demonstrating that CD structure governs both the extent and the trajectory of pigment transformation during wine aging.

## 4 Conclusions

This study reveals CD–driven modulation of anthocyanin transformations and wine color evolution, offering a mechanistic coloromics framework for predictable control of wine color dynamics. The key findings are summarized as follows:

1) Anthocyanin dynamics and wine color evolution are modulated by CD cavity size and flexibility. α-CD accelerates monomer conversion into pyranoanthocyanin derivatives, enhancing color quality, whereas β-CD and HP-β-CD stabilize monomers and acylated anthocyanins, promoting controlled derivative formation and sustaining color stability.
2) CDs regulate anthocyanin-derived pigment evolution by selectively stabilizing phenolics, with β-CD and HP-β-CD protecting larger or hydrophobic compounds to preserve later derivatives, while α-CD promotes early polymerization of small monomers.
3) Multivariate analyses indicate that early color is driven by monomeric anthocyanins, whereas long-term color stability relies on high-molecular derivatives and pyranoanthocyanin–flavonoid networks, with CDs modulating the kinetics and balance of these competing pathways.
4) CD addition offers a novel strategy to regulate wine color evolution, and the cavity-size-dependent differences among CD types reveal that α-CD and β-CD/HP-β-CD operate through distinct mechanisms to achieve complementary color outcomes. These findings suggest that CD type selection can be tailored to specific winemaking objectives, whether prioritizing early chromatic enhancement or long-term color stability. This study, for the first time, applies mechanistic coloromics to link anthocyanin transformations, derivative accumulation, and macroscopic color evolution, showing how CD structure and selective phenolic stabilization modulate wine color quality.

## CRediT authorship contribution statement

**Caiyun Liu:** Writing – review & editing, Writing – original draft, Validation, Supervision, Software, Resources, Methodology, Investigation, Formal analysis, Data curation, Conceptualization. **Zengshuai Zhang:** Software, Resources, Data curation. **Jing Li:** Validation, Supervision. **Xiaoyu Zhang:** Software, Resources. **Mario Prejanò:** Resources, Methodology. **Tiziana Marino:** Supervision, Software, Resources. **Yongsheng Tao:** Investigation, Funding acquisition. **Yunkui Li:** Writing – original draft, Funding acquisition, Data curation, Conceptualization.

## Ethical approval

This article does not contain any studies with human participants performed by any of the authors.

## Declaration of competing interest

The authors declare that they have no known competing financial interests or personal relationships that could have appeared to influence the work reported in this paper.

## Data Availability

Data will be made available on request.
