# Supplementary material for: Cyclodextrin-mediated improvement of red wine color quality dissected by coloromics and anthocyanin evolution dynamics
Source: Food Chem X. 2026 Jul 14;38:104199. doi: 10.1016/j.fochx.2026.104199 (PMC13400378; doi:10.1016/j.fochx.2026.104199)
Supplement: Supplementary file 1 — Supplementary material [file mmc1.docx]

**Cyclodextrin-mediated improvement of red wine color quality dissected by coloromics and anthocyanin evolution dynamics**

Caiyun Liu^a^, Zengshuai Zhang^a^, Jing Li^a^, Xiaoyu Zhang^a^, Mario Prejanò^b^, Tiziana Marino^b^, Yongsheng Tao^a^, Yunkui Li^a,*^

a College of Enology, Northwest A&F University, Yangling 712100, China;

b Dipartimento di Chimica e Tecnologie Chimiche, Università della Calabria, Arcavacata di Rende, CS, Italy;

*Corresponding author.

Tel: +86-29-87092107; Fax: +86-29-87092991;

E-mail: ykli@nwsuaf.edu.cn

Table S1 Abbreviations and full names of anthocyanin and their derivatives during wine aging.

| \| Abbreviation \| \| --- \| | Full name |
| --- | --- | --- |
| Pnd-G | Peonidin-3-*O*-glucoside |
| Dnd-G | Delphinidin-3-*O*-glucoside |
| Ptnd-G | Petunidin-3-*O*-glucoside |
| Mvd-G | Malvidin-3-*O*-glucoside |
| Cy-AcG | Cyanidin-3-*O*-acetylglucoside |
| Dp-AcG | Delphinidin-3-*O*-acetylglucoside |
| Ptnd-AcG | Petunidin-3-*O*-acetylglucoside |
| Mvd-AcG | Malvidin-3-*O*-acetylglucoside |
| Dnd-CmG | Delphinidin-3-*O*-coumaroylglucoside |
| Ptnd-CmG | Petunidin-3-*O*-coumaroylglucoside |
| Cy-CmG | Cyanidin-3-*O*-coumaroylglucoside |
| Pnd-CmG | Peonidin-3-*O*-coumaroylglucoside |
| Mvd-CmG | Malvidin-3-*O*-coumaroylglucoside |
| Pnd-CafG | Peonidin-3-*O*-caffeoylglucoside |
| Mvd-CafG | Malvidin 3-*O*-caffeoylglucoside |
| Cy-G-Acet | Cyanidin-3-*O*-glucoside-acetaldehyde |
| Pnd-G-Acet | Peonidin-3-*O*-glucoside-acetaldehyde |
| Dnd-G-Acet | Delphinidin-3-*O*-glucoside-acetaldehyde |
| Ptnd-G-Acet | Petunidin-3-*O*-glucoside-acetaldehyde |
| Mvd-G-Acet | Malvidin-3-*O*-glucoside-acetaldehyde |
| Pnd-AcG-Acet | Peonidin-3-*O*-acetylglucoside-acetaldehyde |
| Dnd-AcG-Acet | Delphinidin-3-*O*-acetylglucoside-acetaldehyde |
| Ptnd-AcG-Acet | Petunidin-3-*O*-acetylglucoside-acetaldehyde |
| Mvd-AcG-Acet | Malvidin-3-*O*-acetylglucoside-acetaldehyde |
| Cy-CmG-Acet | Cyanidin-3-*O*-coumaroylglucoside-acetaldehyde |
| Pnd-CmG-Acet | Peonidin-3-*O*-coumaroylglucoside-acetaldehyde |
| Dnd-CmG-Acet | Delphinidin-3-*O*-coumaroylglucoside-acetaldehyde |
| Ptnd-CmG-Acet | Petunidin-3-*O*-coumaroylglucoside-acetaldehyde |
| Mvd-CmG-Acet | Malvidin-3-*O*-coumaroylglucoside-acetaldehyde |
| Pnd-CafG-Acet | Peonidin-3-*O*-caffeoylglucoside-acetaldehyde |
| Mvd-CafG-Acet | Malvidin-3-*O*-caffeoylglucoside-acetaldehyde |
| Pnd-G-Pyr | Peonidin-3-*O*-glucoside-pyruvic acid |
| Dnd-G-Pyr | Delphinidin-3-*O*-glucoside-pyruvic acid |
| Ptnd-G-Pyr | Petunidin-3-*O*-glucoside-pyruvic acid |
| Mvd-G-Pyr | Malvidin-3-*O*-glucoside-pyruvic acid |
| Pnd-AcG-Pyr | Peonidin-3-*O*-acetylglucoside-pyruvic acid |
| Dnd-AcG-Pyr | Delphinidin-3-*O*-acetylglucoside-pyruvic acid |
| Ptnd-AcG-Pyr | Petunidin-3-*O*-acetylglucoside-pyruvic acid |
| Continued Table S2 | |
| \| Abbreviation \| \| --- \| | Full name |
| Mvd-AcG-Pyr | Malvidin-3-*O*-acetylglucoside-pyruvic acid |
| Pnd-CmG-Pyr | Peonidin-3-*O*-coumaroylglucoside-pyruvic acid |
| Dnd-CmG-Pyr | Delphinidin-3-*O*-coumaroylglucoside-pyruvic acid |
| Ptnd-CmG-Pyr | Petunidin-3-*O*-coumaroylglucoside-pyruvic acid |
| Mvd-CmG-Pyr | Malvidin-3-*O*-coumaroylglucoside–pyruvic acid |
| Pnd-CafG-Pyr | Peonidin-3-*O*-caffeoylglucoside–pyruvic acid |
| Dnd-CafG-Pyr | Delphinidin-3-*O*-caffeoylglucoside–pyruvic acid |
| Ptnd-CafG-Pyr | Petunidin-3-*O*-caffeoylglucoside–pyruvic acid |
| Mvd-CafG-Pyr | Malvidin-3-*O*-caffeoylglucoside–pyruvic acid |
| Pnd-G-VPh | Peonidin-3-*O*-glucoside-4-vinylphenol |
| Mvd-G-VPh | Malvidin-3-*O*-glucoside-4-vinylphenol |
| Pnd-AcG-VPh | Peonidin-3-*O*-acetylglucoside-4-vinylphenol |
| Ptnd-AcG-VPh | Petunidin-3-*O*-acetylglucoside-4-vinylphenol |
| Mvd-AcG-VPh | Malvidin-3-*O*-acetylglucoside-4-vinylphenol |
| Pnd-CmG-VPh | Peonidin-3-*O*-coumaroylglucoside–4-vinylphenol |
| Ptnd-CmG-VPh | Petunidin-3-*O*-coumaroylglucoside-4-vinylphenol |
| Mvd-G-PyrVPh | Malvidin-3-*O*-glucoside-pyrano-vinylphenol |
| Cy-G-VG | Cyanidin-3-*O*-glucoside-4-vinylguaiacol |
| Pnd-G-VG | Peonidin-3-*O*-glucoside-4-vinylguaiacol |
| Dnd-G-VG | Delphinidin-3-*O*-glucoside-4-vinylguaiacol |
| Ptnd-G-VG | Petunidin-3-*O*-glucoside-4-vinylguaiacol |
| Mvd-G-VG | Malvidin-3-*O*-glucoside-4-vinylguaiacol |
| Mvd-AcG-VG | Malvidin-3-*O*-acetylglucoside-4-vinylguaiacol |
| Ptnd-CmG-VG | Petunidin-3-*O*-coumaroylglucoside–4-vinylguaiacol |
| Mvd-CmG-VG | Malvidin-3-*O*-coumaroylglucoside–vinylguaiacol |
| Ptnd-CmG-VC | Petunidin-3-*O*-coumaroylglucoside–4-vinylcatechol |
| Mvd-CmG-VPh | Malvidin-3-*O*-coumaroylglucoside–4-vinylphenol |
| Mvd-CafG-VPh | Malvidin-3-*O*-caffeoylglucoside–4-vinylphenol |
| Cat–Pnd-G | (Epi)catechin–peonidin-3-*O*-glucoside |
| GCat–Pnd-G | Gallocatechin–Peonidin-3-*O*-glucoside |
| Cat–Mvd-G | (Epi)catechin–Malvidin-3-*O*-glucoside |
| GCat–Mvd-G | (Epi)gallocatechin-Malvidin-3-*O*-glucoside |
| Cat–Mvd-AcG | (Epi)catechin–Malvidin-3-*O*-acetylglucoside |
| GCat–Mvd-AcG | (Epi)gallocatechin–Malvidin-3-*O*-acetylglucoside |
| GCat–Mvd-CafG | (Epi)gallocatechin–Malvidin-3-*O*-caffeoylglucoside |
| Mvd-G-V-Cat | Malvidin-3-*O*-glucoside-4-vinyl-(epi)catechin |
| Mvd-AcG-V-Cat | Malvidin-3-*O*-acetylglucoside-4-vinyl-(epi)catechin |
| Mvd-CmG-V-Cat | Malvidin-3-*O*-coumaroylglucoside-4-vinyl-(epi)catechin |
| Ptnd-G-EtCat | Petunidin-3-*O*-glucoside-8-ethyl-catechin |
| Mvd-G-EtCat | Malvidin-3-*O*-glucoside-8-ethyl-(epi)catechin |
| Mvd-G-Dim | Malvidin-3-*O*-glucoside dimer |
